# Stochastic Stabilization of Phenotypic States: The Genetic Bistable Switch as a Case Study

**DOI:** 10.1371/journal.pone.0073487

**Published:** 2013-09-11

**Authors:** Marc Weber, Javier Buceta

**Affiliations:** Computer Simulation and Modelling (Co.S.Mo.) Lab, Parc Científic de Barcelona, Barcelona, Spain; Universitat Politecnica de Catalunya, Spain

## Abstract

We study by means of analytical calculation and stochastic simulations how intrinsic noise modifies the bifurcation diagram of gene regulatory processes that can be effectively described by the Langevin formalism. In a general context, our study raises the intriguing question of how biochemical fluctuations redesign the epigenetic landscape in differentiation processes. We have applied our findings to a general class of regulatory processes that includes the simplest case that displays a bistable behavior and hence phenotypic variability: the genetic auto-activating switch. Thus, we explain why and how the noise promotes the stability of the low-state phenotype of the switch and show that the bistable region is extended when increasing the intensity of the fluctuations. This phenomenology is found in a simple one-dimensional model of the genetic switch as well as in a more detailed model that takes into account the binding of the protein to the promoter region. Altogether, we prescribe the analytical means to understand and quantify the noise-induced modifications of the bifurcation points for a general class of regulatory processes where the genetic bistable switch is included.

## Introduction

Cells’ functions are controlled by networks of interacting genes and proteins that set the basis of regulation, signaling and response. Over the past decade a number of studies have shown that the level and activity of the species involved in such regulatory circuits fluctuate [1]. These fluctuations are mainly due to the inherent randomness of biochemical reactions that becomes especially significant when the number of molecules of the chemical species is very low [2]. Biochemical noise, either intrinsic or extrinsic, is not necessarily a nuisance but an essential biological component that in many situations has a positive functional role [3], as for example improving cellular regulation [4].

Importantly, stochastic effects are believed to play also an important role in cell differentiation [5]. Thus, noise allows cells that are exposed to the same environment to choose between different fates, thereby increasing the phenotypic diversity. In this regard, the simplest, non-trivial, regulatory system showing phenotypic multi-stability correspond to a genetic switch with two possible stable solutions: low/high concentrations of a regulatory protein. The core of the genetic circuit underlying bistable systems typically involves a protein that up-regulates its own production, leading to a positive feedback loop. Such a behavior has been found in a number of biological systems, as for example the lactose utilization network in *E. coli* [6], and has been also implemented in synthetic circuits [7]–[9]. Consequently, the characterization of genetic switches is important both for the development of larger and more robust synthetic circuits that use small gene modules with well-defined behaviors [10] and for the understanding of complex processes such as cell differentiation.

The conceptual framework of cell differentiation is rooted in Waddington’s ideas about the projection of the genotype space into the phenotype counterpart [11]. Therein phenotypes are associated with attractors, i.e. stable fixed points, in a phase space (the epigenetic landscape) that can be parametrized by the concentration of the molecular species of interest (genotype) [12]. Interestingly, several studies have shown that a stochastic bifurcation diagram (i.e. a stochastic epigenetic landscape) can differ significantly from its deterministic counterpart [13]–[17]. Recent advances in the field include the noise-induced bimodality in the response of the ERK signaling pathway [18] or the increased stability of phenotypic states in bistable systems due to noisy contributions [16], [19]. Moreover, recent studies have clarified the role of different noisy sources for defining the global phenotypic attractor in bistable regulatory systems [17]. Still, despite these efforts, there is a lack of a theoretical formalism to easily understand how those changes in the phenotypic stability are driven by the inherent biochemical fluctuations.

Here, we introduce a perturbative theory to analyze how noise modifies the epigenetic landscape. In particular, we address the problem of the stochastic stabilization/destabilization of a phenotypic state with respect to the noise-free system. We illustrate this phenomenon by means of the well-characterized example mentioned above: a genetic bistable switch. Our results show that noise stabilizes, and consequently favors, one phenotypic landscape with respect to the deterministic system. In addition, we examine the role played by biochemical fluctuations with a non-null correlation time and show that, while the effect is lessened, the stochastic modification of the epigenetic landscape also emerges. Our theoretical calculations are generic and can be applied to any regulatory circuit that is susceptible to be described by the Langevin formalism and in particular to a general class of regulatory processes with feedback where the genetic switch is included. Moreover, in order to check that our conclusions are not an artifact due to an oversimplified mathematical description, we demonstrate that the effect also develops in a detailed model of the genetic switch that we simulate by means of the Gillespie algorithm.

The paper is organized as follows. In the Methods section, we introduce our theoretical approach to analyze the stochastic modification of the epigenetic landscape for a general class of regulatory processes. In the results section, we first apply our finding to a simple model describing a genetic switch. Subsequently, by means of numerical simulations of a more detailed model, we demonstrate that the stochastic stabilization effect is generic for this kind of architecture. Finally, in the Discussion section, we present the main conclusions and discuss about the applicability and relevance of our study.

## Methods

### Stochastic Modification of Bifurcation Points: Perturbative Theory

In the context of genetic circuits, a definition of stochastic bifurcation has been previously proposed, based on experimental data [20] or results from gene network models [13], [15]. In general, a stochastic bifurcation is characterized by a qualitative change in one of the observables of the stochastic process. In the case of a bistable system, one may consistently identify two subpopulations of cells whose states are distributed around the two stable states (or attractors) [20]. We will follow this approach and define the stochastic system as monostable if its steady state probability distribution is unimodal and bistable if its distribution is bimodal. More complex stochastic bifurcations has been proposed, for example in the case of systems with oscillatory dynamics [15].

In the context of gene regulatory circuits, the chemical kinetics formalism that address the different processes underlying regulation leads to a Master equation representation [21]. The latter can be approximated by different expansion techniques to an Itô Langevin equation for the concentration of the species [21], [22]. Thus, we start by studying a general stochastic system described by a Itô Langevin equation of one variable 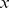 and control parameter 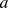:
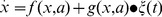
(1)

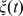
 being a Gaussian white noise such that,



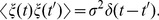
(2)The symbol 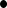 indicates that the stochastic process must be interpreted according to Itô.

Under these conditions, the stationary probability density reads [23],
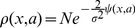
(3)



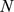
 being a normalization constant and,
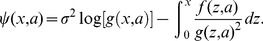
(4)


It is easy to show that the extrema of the probability density are located at points that satisfy
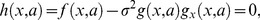
(5)where we have used the compact subindex notation for the partial derivatives, e.g. 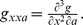


On the other hand, the inflection points of the probability density satisfy
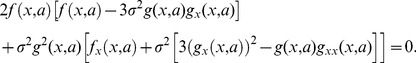
(6)


Thus, if there is a stochastic bifurcation point such that a new extrema appears/disappears, the bifurcation points 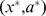 must satisfy equations (5) and (6) simultaneously leading to
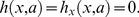
(7)


Notice that in the deterministic case, i.e. 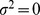, the bifurcation points are given by the points 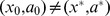, satisfying the equations 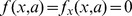.

In order to analyze how the bifurcation points vary with due to the presence of fluctuations, we assume that if the noise intensity is small then the following 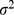-expansion of the points 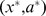 holds,
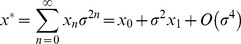
(8)

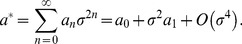
(9)


Thus, by expanding 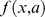 and 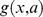 in powers of 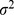 and collecting terms, the equations (7) read
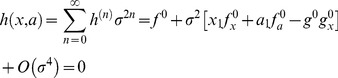
(10)

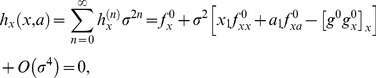
(11)where the superindex 0, indicates that the functions are evaluated at the point 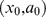. By solving the conditions 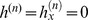, we can obtain the corrections 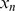 and 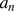 up to an arbitrary order 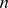. As expected, at zero order, 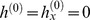, we obtain the deterministic conditions for the bifurcation point: 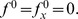 At order one, 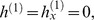 we get



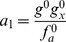
(12)

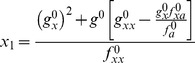
(13)


We have also calculated the corrections at order 2, yet, the expressions are cumbersome and we provide the results in the Supplementary Information (see Text S1).

Our formalism and results can be applied to a general class of regulatory processes with feedback described by the following biochemical reactions:
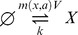
(14)where 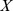 stands for the regulatory species (number of molecules), 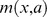 is the gene regulatory function describing effective production (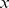 being the concentration of 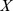), 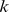 the degradation rate and 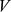 the cellular volume. It is easy to demonstrate that in this case,



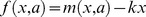


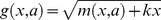


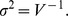



A bifurcation point leading to multistability will exist if there are at least three non-negative real solutions satisfying the equation 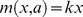. In those cases, the modification of the bifurcation points of the deterministic system due to the biochemical noise reads
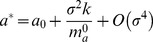
(15)

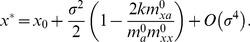
(16)


While in terms of 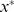 it is not trivial to envision the sign of the displacement caused by noise, in terms of 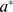 it is easy at least at first order: its sign is prescribed by the putative role played by 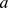 in the regulation of species 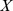. That is, if 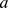 promotes positive regulation (activator), 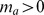, then 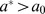, as in the case of the auto-activating genetic switch (see below). Contrariwise, if 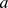 is an inhibitor of production then noise will advance the location of the bifurcation point (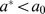).

### Birth and Death Process: Exact Solution

The formalism and results presented above, apply to the (approximated) Itô Langevin description. Yet, the exact solution of the regulatory processes described by (14) can be also obtained. Thus, by comparing the exact epigenetic landscape with that resulting from the Langevin description we can validate the scope of our calculations beyond the numerical simulations. Note that the biochemical reactions (14) are equivalent to the birth and death processes [24]:
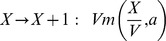


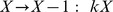



The master equation describing this process is.
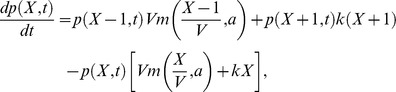
where 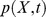 stands for the probability of having 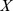 number of molecules at time 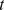. By imposing that at equilibrium the net flux between neighboring states becomes null (detailed balance), the stationary probability, 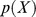, reads,
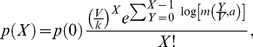
(17)where 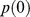 is a normalization constant such that 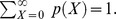


### Non-null Memory Fluctuations

Single cell level experiments have revealed that intrinsic fluctuations show a “short” correlation time, i.e. white-noise-like [25]. Yet, the white noise is an idealization about the actual behavior of fluctuations since implies a memoryless process. In order to clarify the consequences of this fact in regard to modification of the epigenetic landscape, we also examine the role play by colored fluctuations. The so-called Ornstein-Uhlenbeck (OU) process is defined by the stochastic differential equation [23]:
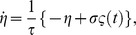
where 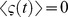 and 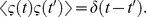 Under these conditions the mean and the correlation of the OU process read,



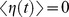


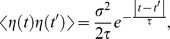
where 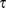 stands for the correlation time (memory) of the fluctuations. In the limit 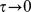 the OU process tends to a white noise with intensity 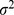, that is,



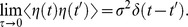



Importantly, the Wong-Zakai theorem [23] shows that when 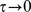, and the OU process is multiplicative in the stochastic differential equation, the right interpretation of the latter is Stratonovich instead of Itô. In this regard, note that the stochastic differential equation,
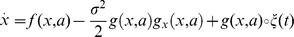


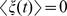


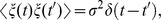
where 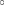 indicates that the stochastic integral must be interpreted according to Stratonovich, represents the same stochastic process as Eq. (1). Consequently, the stochastic differential equation,
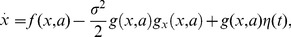
(18)where 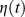 stands for the OU process, has the same solution as Eq. (1) in the limit 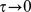 and the modification of the epigenetic landscape is the same as in the white noise case (data not shown).

Equation (18) cannot be solved analytically for an arbitrary value of 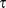. However, we can elucidate the modification of the epigenetic landscape in the limit 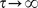, that is, for long correlation times. We notice that in that limit, for a finite noise intensity, the OU process vanishes and the system behaves as the deterministic system [23],
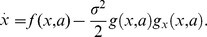



Therefore, for long correlation times, the bifurcation points are located at points 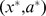 satisfying,
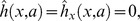
where,



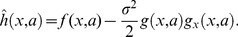
(19)Interestingly, these are the same conditions as (7) if in Eq.(5) 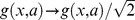 (alternatively if the noise intensity is halved). Thus, at order one, for long correlation times, the correction of the bifurcation points read,
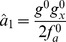
(20)

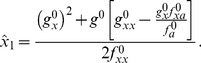
(21)


In summary, as the correlation time of the fluctuation increases, the shift effect over the bifurcation points is lessened (see numerical simulation results below). Yet, even in the limit case of infinite memory fluctuations a shift appears and its sign does not depend on the colored character of the noise.

## Results

**Figure 1:**
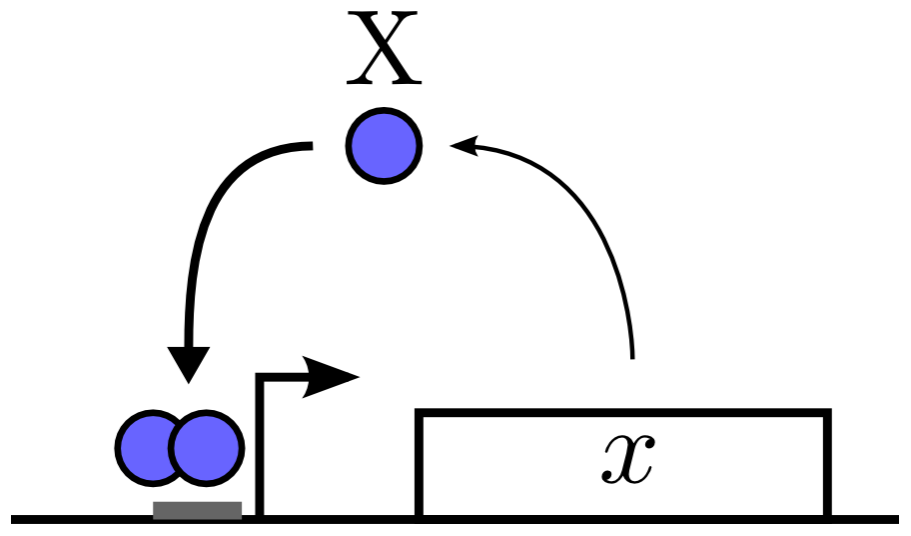
Scheme of the genetic auto-activating switch model. The expression of gene 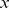 leads to protein 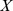 that after oligomerization binds to its own promoter acting as an self-activator.

We now apply our theoretical calculations to a well-characterized system: the auto-activating switch [6], [8]. In this genetic circuit, a protein forms an oligomer that binds to the promoter region of its own gene and activates its expression (see Figure 1). As shown elsewhere, this regulatory process can be effectively described by the Hill function formalism and leads to the following deterministic equation for the concentration, 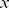, of protein [16]:
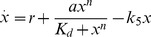
(22)where 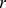 is the basal expression rate (promoter leakiness), 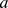 the maximum production rate (efficiency of the auto-activation), 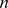 the cooperativity (oligomerization index), 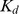 the concentration of protein yielding half-maximum activation and 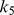 the degradation rate. Notice that the auto-activating regulatory scheme fits within the general class (14): 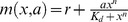 being the gene regulatory function. Alternatively, the dimensionless version of eq. (22) reads
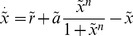
(23)with 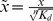, 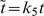, 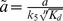, 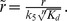


If 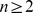 and 
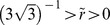
 then the system exhibits a bistable behavior (phenotypic variability) for a range of values of 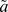. Here we choose 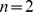 and 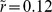. Notice that the system has two bifurcation points that define the bistability region. These points correspond to the solutions 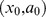 of the polynomial equations,
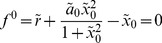
(24)

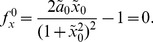
(25)


We now examine how the bifurcation diagram, the epigenetic landscape, changes due to the biochemical fluctuations. In particular, we look at the shift of the bifurcation points. The It Langevin equation that corresponds to this system reads [16], [22]

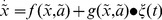
(26)

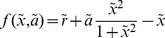
(27)

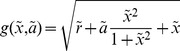
(28)

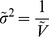
(29)where 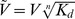 is the dimensionless volume. By proceeding as described in the previous section, see eqs. (15) and (16), the bifurcation points 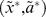 read



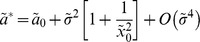


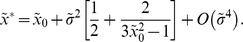



**Figure 2:**
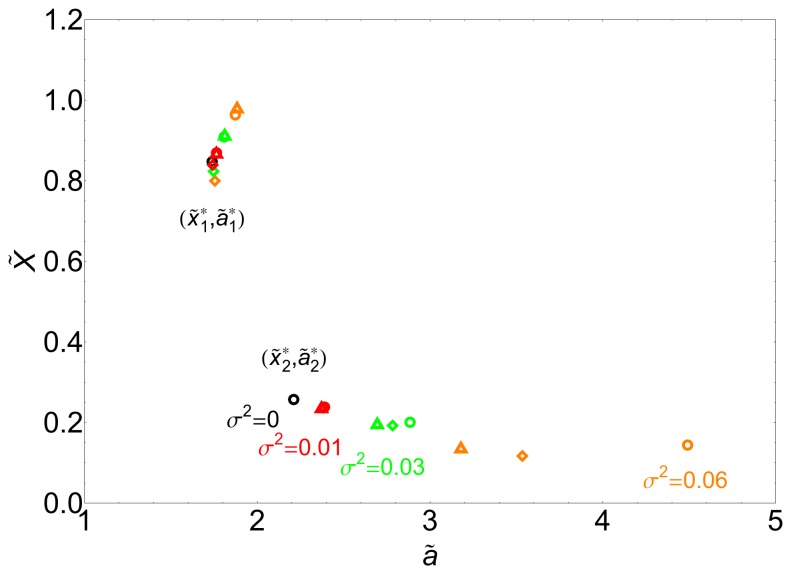
Noise-induced shift of the bifurcation points for the simple genetic switch. Change in the position of the bifurcation points 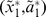 and 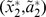 for different values of noise intensity: 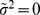 (black symbols), 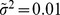 (red symbols), 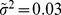 (green symbols) and 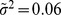 (orange symbols). Numerical exact solution (circles), first order approximation (triangles) and second order approximation (diamonds). The biochemical fluctuations shift the position of both bifurcation points but the effect for 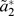 is more noticeable and widens the bistability region that ultimately promotes the stability of one phenotype.

**Figure 3:**
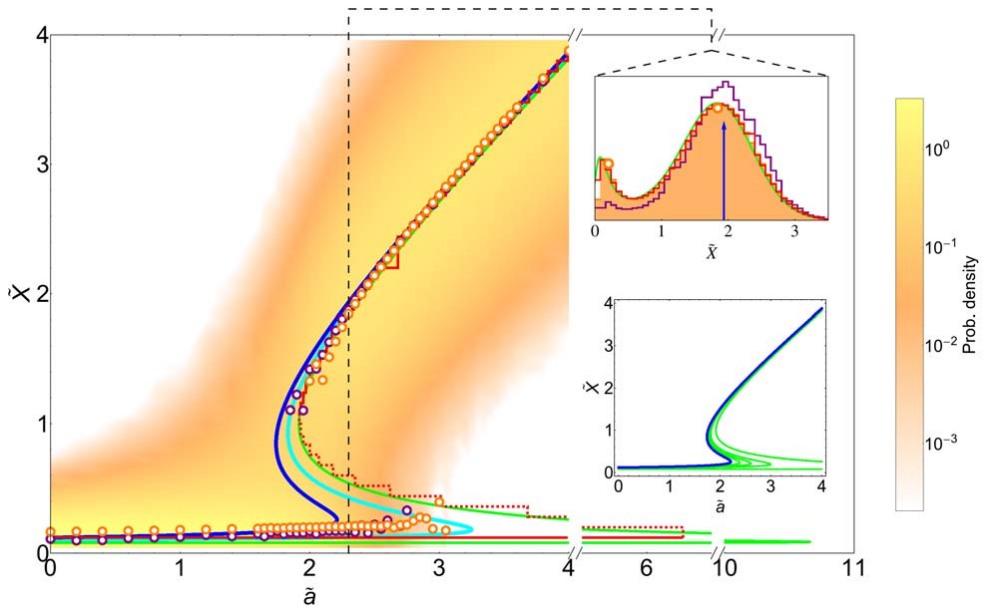
Bifurcation diagram of the simple genetic switch. Deterministic system (blue line), white-noise stochastic system (Langevin: green line; Exact solution: red line). The bifurcation diagram of a system with colored fluctuations in the limit 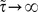 is also depicted (cyan line). In all cases 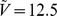. The results from stochastic simulations are in agreement with the analytical results, as can be seen by the detected peaks (orange circles) of the probability distribution of 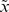 at steady state (color code, logarithmic scale). The numerical simulations for a non-null correlation time noisy sytem, 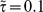, indicate that the effect is lessened when memory is considered (purple circles). The top inset reveals that the probability distributions obtained in numerical simulations (orange histogram) are in perfect agreement with the exact solution (red line) and the Langevin description (green line), 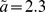. For that value of the control parameter the deterministic system only have one stable solution and the probability distribution corresponds to a Dirac delta (blue arrow). When the correlation time of the noise is not null, 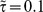, the stability of the low state decreases with respecto to the white noise case (purple line). The circles in the inset denote the maxima as detected by the Gaussian peak detection algorithm. Bottom inset: increasing noise (decreasing volume) clearly extends the stable branch of the low state, an effect that we call *stochastic stabilization*: 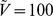, 50, 30 and 12.5.

The analytical expressions for the second order corrections are provided in the Supplementary Information (see Text S1). Figure 2 shows, in agreement with our analytical calculations, the location of the bifurcation points as a function of the noise intensity 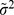, at first (triangles) and second (diamonds) orders as well as the exact solutions (circles) (see also Figure 3 bottom inset). Note that in terms of 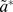 the correction due to the noise is always positive. Consequently both bifurcation points, those defining the bistable region, are shifted to the right. Moreover, the shift largely increases as 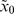 approaches to zero, and the bistable region widens with respect to the deterministic system. In addition, the low state, for which 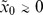, has a negative correction in terms of 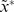, i.e. 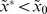. Altogether, our calculations indicate that one of the states (the low protein concentration one) becomes more stable due to the biochemical noise. We call this effect the *stochastic stabilization* of a phenotypic state. Alternatively, this phenomenon can be interpreted as a noise-induced bistability since there is a range of values of the control parameter for which the stochastic system displays a bistable behavior in opposition to the monostable response of the deterministic system.

Figure 3 shows the analytical bifurcation diagrams for the deterministic and the stochastic cases. In the stochastic cases, with regard to the Langevin approximation, we define the stable and unstable branches by means of the extrema of the probability distribution, i.e. by numerically solving the condition (5) and, in the case of the exact analytical solution, Eq. 17, by numerically finding the extrema of 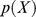. The results support the stochastic stabilization phenomenon: compared with the deterministic system the low protein concentration state becomes stable for a larger range of values of the control parameter as the noise intensity increases. Moreover, our results validate the Langevin approximation since is in good agreement with the exact solution.

In order to gain more insight into the stabilization phenomenon, we perform stochastic simulations of equations (1) and (18). In these cases, the position of the maxima are computed by using a Gaussian peak detection algorithm over the numerical probability distributions obtained in the simulations. On one hand, in the white noise case, the position of the maxima show a good agreement with the analytical results. However, our simulations reveal that despite noise extends the low state stability to higher values of 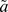, the probability of residing at the low state quickly drops when increasing the control parameter. This is the reason why the detected peaks of the low state from the simulations do not extend as far as the stable branch of the low state computed from the analytical calculations. On the other hand, in the case of the colored noise (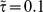), the simulations confirm the analytical calculations when the biochemical fluctuations are not memoryless: for the same noise intensity of the white noise case, the effect is lessen, yet present and the low state becomes stabilized.

The noise-induced bistable behavior is revealed by the bimodal shape of the stationary probability density in opposition to the deterministic system for which the only stable solution is the high state (Figure 3 top inset). The analytical distribution computed from (3) agrees with both the exact solution and the distribution computed from the stochastic simulations. Notice that when colored noise is considered the stochastic stabilization effect is lessened as revealed by the drop of the maximum that correspond to the low state.

**Figure 4:**
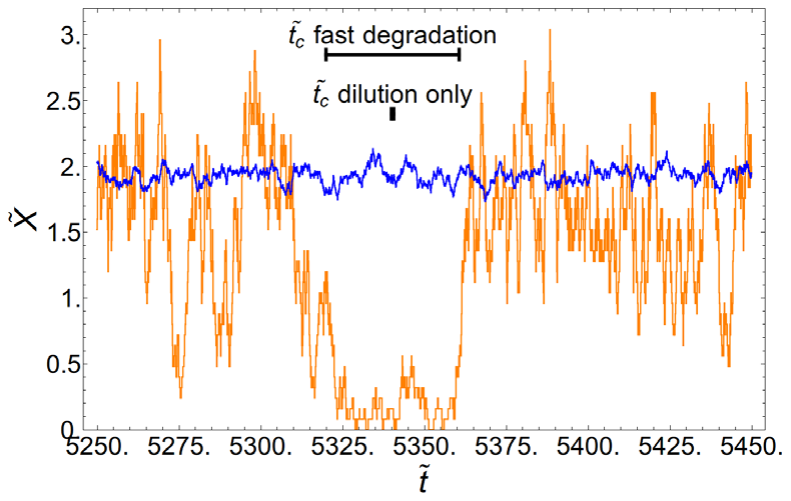
Trajectories for the simple genetic switch i for small and large volume. Trajectories from stochastic simulations of the simple genetic switch model for small volume 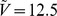 (orange line) and large volume 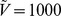 (blue line) for 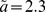. For this value of parameter 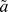, the system is monostable and stays at the high state (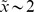) when the noise intensity is small (large volume), while it is bistable when the noise intensity is high (small volume) and jumps between the low state (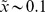) and the high state (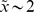). The black bars indicate the characteristic duration of a bacterial cell cycle (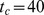 minutes) under two different conditions of the protein degradation rate and show that the residence time at the low state is large enough to be noticeable as a phenotype: stable proteins driven by dilution effects (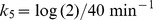) and unstable proteins with fast degradation induced by tagging (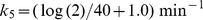).

Figure 4 shows stochastic trajectories for 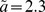 when the fluctuations are white. At this value, the low state is unstable in the deterministic system and the only plausible phenotype is the high state. Indeed, when the volume is large (low noise intensity) the system is monostable and stays at the high state (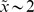). However, when the volume is small (high noise intensity) the system exhibits a bistable behavior, jumping between the high state and the low state (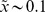). In order to show that the residence time at the low state is large enough and, as a result, noticeable as a phenotype, we indicate in Figure 3 the characteristic duration of a bacterial cell cycle (40 minutes) under two different conditions of the protein degradation rate: stable proteins with an effective degradation driven by the cellular growth (dilution), 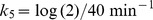, and unstable proteins with fast degradation induced by tagging [26], 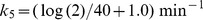.

Our results towards the understanding of the modification of the phenotypic landscape due to the biochemical fluctuations are based on a simplified view of the regulatory process (the genetic switch) described by a single variable. However, one might wonder if our results are applicable when a more detailed model is considered, that is, if our predictions are an artifact due to an oversimplified mathematical description. In order to address this question, we consider a more detailed, yet equivalent, model of the genetic switch using the chemical kinetics formalism. In particular, our model takes into account the basal expression rate, the binding/unbinding events of the protein oligomer to the promoter, an effective transcription/translation rate, and the protein degradation:
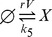
(30)

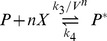


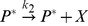
(31)where 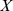 stands for the protein (number of molecules), 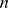 its oligomerization index and 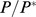 the unbound/bound states of the promoter. These reactions lead to the following deterministic description in terms of ordinary differential equations describing the concentration of chemical species,



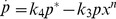
(32)

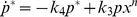
(33)

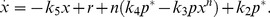
(34)


Binding/unbinding events are fast reactions compared to the protein production and the degradation, i.e. 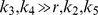. Thus, a quasi-steady state approximation can be implemented such that 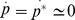. The latter combined with the conservation law of the total promoter concentration, 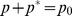, leads to the following equation,
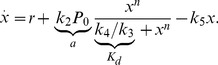
(35)


Therefore, assuming that the binding/unbinding of the protein to the DNA are fast reaction, this model leads to the same deterministic equation as in the simple genetic switch model. However, its stochastic description in terms of the set of equation (30–31) is far more complex that equation (26) even considering that binding/unbinding are fast events since 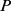 and 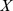 are correlated quantities and each species exhibits a fluctuating dynamics [17]. Then, we perform stochastic simulations of equations (30–31) using the Gillespie algorithm [27] and apply the peak detection method to elucidate the bifurcation changes in the epigenetic landscape.

**Figure 5:**
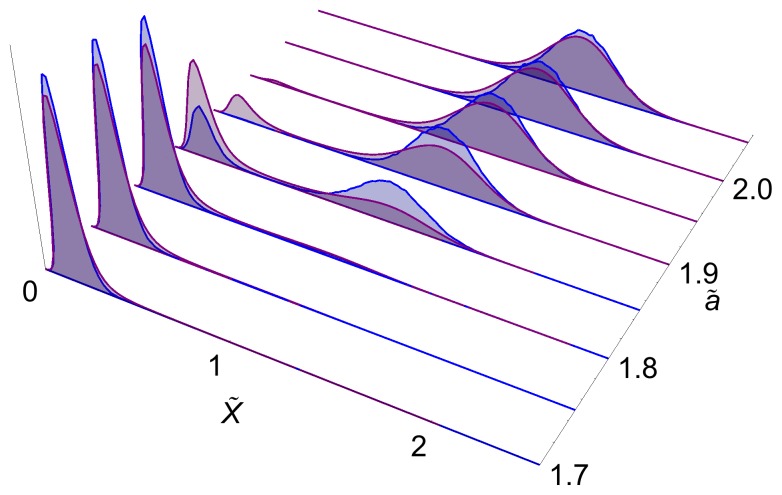
Steady state probability distribution for the simple and detailed models. Probability distribution at steady state for volume 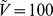 for the simple (purple filled curves) and the detailed (blue filled curves) genetic switch models, for different values of the control parameter 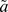. The distributions match well except in the region where distributions are bimodal and highlight the fact that while the deterministic description is the same in both models, the stochastic one is not.

**Figure 6:**
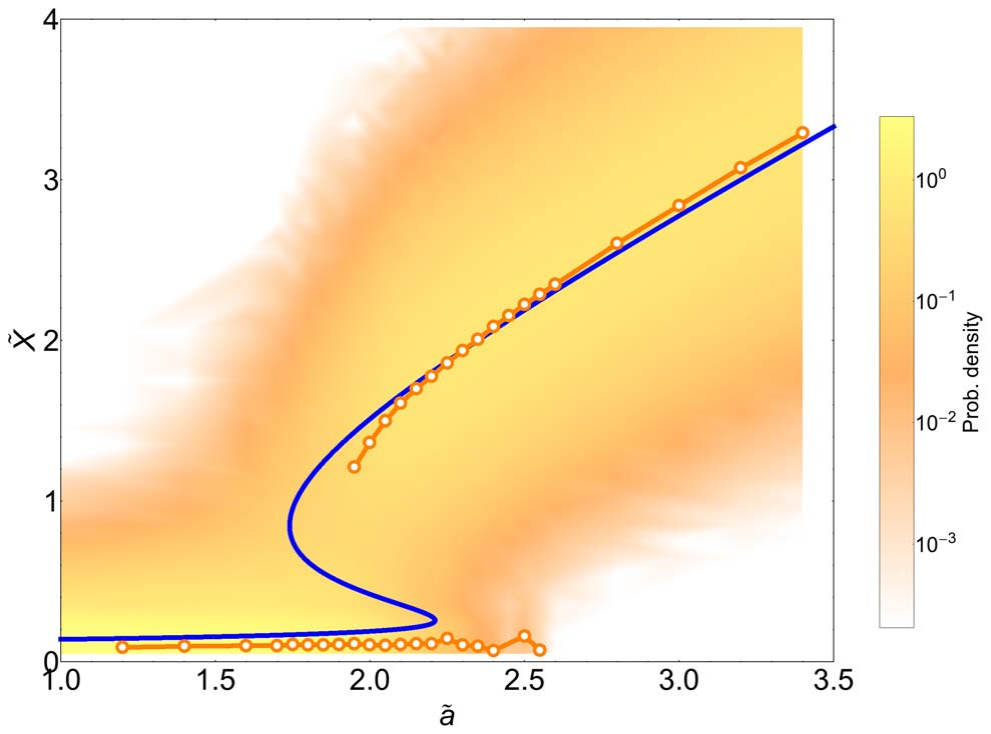
Bifurcation diagram of the detailed genetic switch. Bifurcation diagram of the detailed genetic switch with fast protein-DNA binding/unbinding, The results from stochastic simulations at low volume 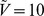 shows that noise induces the same stochastic stabilization effect as in the simple genetic switch model, as can be seen by the position of the peaks of the distribution from the simulations (orange circles) compared to the deterministic system (blue solid line). Color code denotes the probability distribution from stochastic simulations for 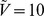 in logarithmic scale.

In order to reduce the number of parameters, we use the same definition of dimensionless variables as above. Compared to the simplified model, the detailed model has two additional parameters, 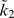 and 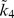. Parameter 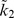 is related to the control parameter 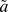 by the relation 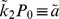. In order to change 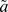, we vary the value of 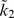 and keep fixed the DNA copy number 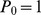. In our simulations the value of 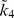 is fixed (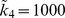) and ensures that equation (35), and consequently the deterministic bifurcation diagram, applies when fluctuations are neglected. Yet, when considering the noise, the differences between the simple and the detailed stochastic model are noticeable by examining the stationary probability distributions (see Figure 5). Nonetheless, in agreement with our theoretical approach, the bifurcation diagram, Figure 6, shows that noise promotes the stability of the low state compared to the deterministic system. However, the stochastic stabilization effect is smaller than in the simple genetic switch model. For example, for a volume 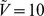 the maximum in the distribution corresponding to the low state can be detected up to the value 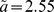 for the detailed model, while in the simple model it can be detected up to 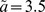.

## Discussion

By using the auto-activating genetic switch as a case study, we have shown that the biochemical intrinsic noise may induce a shift in the position of the bifurcation points such that the region of parameter values for which the stationary probability distribution is bimodal increases with fluctuations with respect to the deterministic situation. In particular, the low state stability is extended; an effect that we call *stochastic stabilization* and that we have shown that, in essence, does not depend on the colored character of the fluctuations. The perturbative method that we have introduced is general and can be applied to any stochastic system describing a gene regulatory network. Yet, we point out that the method is limited to the case of one-dimensional stochastic differential equations for which the general solution of the stationary probability density can be written explicitly. Nonetheless, we have shown by means of simulations of a more detailed model, that the stochastic stabilization phenomenon does not depend on this particular detail thus suggesting a generic phenomenon in positive feedback switches (see for instance [19]). Previous studies [14], [15] have also found that noise changes the position and even the number of stable states [18]. In this regard, our study provides a theoretical framework to predict and understand such phenomenology.

The results of the detailed model differ quantitatively from the simple model when fluctuations are considered. In particular, the probability distributions and the range of values for which the latter are bimodal are different. Thus, although the deterministic descriptions of both models are totally equivalent (as long as the quasi-steady state approximation holds) this is not true when considering the biochemical fluctuations. In fact, the dynamics at steady state are quite different and we find that in the detailed model the switching rate from the low to the high state is slower than in the simplified model (data not shown). These results are in agreement with other studies about genetic switches: for example in the case of the genetic toggle switch it has been shown that protein-protein interactions can be safely eliminated (adiabatically) but protein-DNA interactions, even though are also fast, lead to noticeable changes in the switching rates if neglected [28]. It is also interesting to place our findings in the context of the role played by different noisy sources, gene switching, translational and transcriptional, for defining the global attractor of bistable systems [17]. Thus, it has been recently shown that the modulation of the intensity of these fluctuations can actually condition the global attractor (the most represented phenotype) and that the elimination of gene switching noise by means of the adiabatic approximation can supress some phenotypes. Our results are consistent with this sudy and provide further means to analyze and understand such phenomenology.

In this study, we have modulated the intensity of intrinsic fluctuations keeping the same concentration and varying the volume of the system. One may wonder how the number of proteins in our model and the intensity of the fluctuations compares with the situation found in real biological systems. The maximum intensity of noise we have used corresponds to a volume of 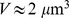 (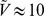), for which the average number of protein 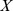 in the low state is 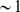. Such volume is in fact the typical volume of an *E. coli* cell [29] and this very low copy number of proteins has been measured in bacteria. For example, the number of LacY repressors in the lactose operon of *E. coli*, an auto-activating genetic switch, has been found to be between 0 and 10, most of the cells having zero or very few molecules [30], and single molecule measurement of 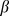-galactosidase in *E. coli* [31] have also reported an average level of 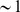 enzyme per cell. Consequently, the number of molecules, and therefore the noise intensity, used in our study is consistent with experimental data on bacterial regulatory networks. Moreover, the intensity of intrinsic noise may be even larger when considering protein expression bursts [32], a phenomenon that we have not included in our modeling.

Summarizing, our study supports the idea that the biochemical noise, far for being a nuisance, is an essential component of genetic regulation and cell functioning. Here we have shown how biochemical noise modifies the location of bifurcation points of the epigenetic landscape with respect to a noise-free system and the impact of this phenomenon for promoting the stability of phenotypic states. We have applied our findings to the well characterized case of the genetic switch that, in its simplest version, belongs to a general class of regulatory processes for which our formalism can be applied. Finally, whether our results are applicable or not to complex fate decision and differentiation processes is a matter of further research. In that regard, fate decisions in some embryonic stem cells are driven by an excitable dynamics that includes positive feedback loops as the one we have considered herein [33]. Hence, we speculate that noise would be also playing a role in redesigning the epigenetic landscape in those cases. Work along this direction is in progress.

## Supporting Information

Text S1 Perturbative theory, second order expansion.
(PDF)
