# Supplementary material for: Stochastic Stabilization of Phenotypic States: The Genetic Bistable Switch as a Case Study
Source: PLoS One. 2013 Sep 11;8(9):e73487. doi: 10.1371/journal.pone.0073487 (PMC3770683; doi:10.1371/journal.pone.0073487)
Supplement: Text S1 — Perturbative theory, second order expansion. (PDF) [file pone.0073487.s001.pdf]

Text S1

1 Perturbative theory, second order expansion

The second-order coefficients of the general expansion read

$$x_2 = \frac{1}{6f_a^3 f_{xx}^3}(-f_{xa}^3 f_{xx} g^2 g_x^2 + f_a f_{xa}^2 g$$
$$g_x(-f_{xxx} g g_x + 6f_{xx} g_x g_{xx} + 2f_{xx} g g_{xxx}) + f_a(-f_{xaa} f_{xx}^2 g^2 g_x^2 +$$
$$f_a(-f_a f_{xxx}(g_x^2 + g g_{xx})^2 + 2f_{xx}^2 g g_x(2g_x g_{xa} + g_a g_{xx} + g g_{xxa})$$
$$- 2f_{xx}(g_x^2 + g g_{xx})(f_{xxa} g g_x - 3f_a g_x g_{xx} - f_a g g_{xxx}))) + f_{xa}$$
$$(f_{aa} f_{xx}^2 g^2 g_x^2 + f_a(2f_a f_{xx}^2 g g_x(g_a g_x + g g_{xa}) + 2f_a f_{xxx} g g_x(g_x^2 + g g_{xx})$$
$$+ f_{xx}(2f_{xxa} g^2 g_x^2 + f_a(g_x^4 - 4g g_x^2 g_{xx}$$
$$- 6g_x^3 g_{xx} + g^2 g_{xx}^2 - 6g g_x g_{xx}^2 - 2g(g_x(g + g_x) + g g_{xx}) g_{xxx})))))$$

$$a_2 = -\frac{1}{6f_a^3 f_{xx}}(-f_{xa}^2 g^2 g_x^2 + f_{aa} f_{xx} g^2 g_x^2 + 2f_a f_{xa} g g_x(3g_x g_{xx} + g g_{xxx}) + f_a^2$$
$$(2f_{xx} g g_x(g_a g_x + g g_{xa}) + (g_x^2 + g g_{xx})(g_x^2 + g g_{xx} - 6g_x g_{xx} - 2g g_{xxx})))$$

In the particular case of the bistable switch the above expressions lead to,

$$x_2 = -(3(1+x_0^2)^6 + 2a_0(1+x_0^2)^2(-12+x_0(7+3x_0(16+x_0(2+x_0(-12+5x_0))))))$$
$$+ a_0^2(1 + x_0(-24 + x_0(2 + 3x_0(8 + 3x_0(6 + 24x_0 - 24x_0^3 - 7x_0^4 + 6x_0^6))))))/$$
$$(12a_0^2 x_0(1 + x_0^2)^2(-1 + 3x_0^2)^3)$$

$$a_2 = -\frac{1}{12a_0 x_0^4(1 + x_0^2)^3(-1 + 3x_0^2)}((1 + x_0^2)^5 + 2a_0 x_0(1 + x_0^2)^2$$
$$(2 + x_0(12 + x_0(1 + 3(-4 + x_0)x_0)))$$
$$+ a_0^2 x_0^2(3 + x_0(24 + x_0(5 + 3x_0(16 - 3x_0(-1 + x_0(8 + x_0)))))))$$
